# Factors Associated With Higher Levels of Grief and Support Needs Among People Bereaved During the Pandemic: Results from a National Online Survey

**DOI:** 10.1177/00302228221144925

**Published:** 2022-12-21

**Authors:** Lucy E. Selman, Damian J. J. Farnell, Mirella Longo, Silvia Goss, Anna Torrens-Burton, Kathy Seddon, Catriona R. Mayland, Linda Machin, Anthony Byrne, Emily J. Harrop

**Affiliations:** 1 Palliative and End of Life Care Research Group, Population Health Sciences, Bristol Medical School, 1980 University of Bristol, Bristol, UK; 2 School of Dentistry, 2112 Cardiff University, Cardiff, UK; 3 Marie Curie Research Centre, 2112 Cardiff University, Cardiff, UK; 4 PRIME Centre, Division of Population Medicine, 2112 Cardiff University, Cardiff, UK; 5 606975 Wales Cancer Research Centre, Cardiff, UK; 6 Department of Oncology and Metabolism, 7315 University of Sheffield, Sheffield, UK; 7 4212 Keele University, Keele, UK

**Keywords:** Bereavement, grief, pandemics, coronavirus infections, baseline survey, bereavement care

## Abstract

We identified factors associated with higher levels of grief and support needs among 711 people bereaved during the COVID-19 pandemic in the UK (deaths 16 March 2020-2 January 2021). An online survey assessed grief using the Adult Attitude to Grief (AAG) scale, which calculates an overall index of vulnerability (IOV) (range 0–36), and practical and emotional support needs in 13 domains. Participants’ mean age was 49.5 (SD 12.9); 628 (88.6%) female. Mean age of deceased 72.2 (SD 16.1). 311 (43.8%) deaths were from confirmed/suspected COVID-19. High overall levels of grief and support needs were observed; 28.2% exhibited severe vulnerability (index of vulnerability ≥24). Grief and support needs were higher for close relationships with the deceased (vs. more distant) and reported social isolation and loneliness (*p* < 0.001), and lower when age of deceased was above 40–50. Other associated factors were place of death and health professional support post-death (*p* < 0.05).

## Introduction

Over 6.4 million people have died of COVID-19 globally (as of July 2022), leaving over 57.6 million people bereaved (Verdery et al., 2020). Pandemic infection control restrictions have had a detrimental impact on end-of-life and bereavement experiences, regardless of cause of death. Lack of contact with family and friends at the time of death and funeral restrictions have been particularly distressing (Hanna et al., 2021; Torrens-Burton et al., 2022). Studies during (Breen, Lee, & Neimeyer, 2021; Maarten C. Eisma et al., 2021; Mayland et al., 2021) and before the pandemic (Kentish-Barnes et al., 2015; Kristensen et al., 2012) have found that traumatic end-of-life experiences and sudden deaths, which are common in COVID-19, exacerbate the severity of grief experiences. Disruptions to support networks also increase risks of poor bereavement outcomes (Lobb et al., 2010; Smith et al., 2020), although the impact of the pandemic in this regard is not yet fully understood.

To inform clinical practice and bereavement support provision and direct resource allocation, evidence is needed to identify groups potentially at risk of difficulties in their grief and/or high levels of support needs. There is a growing relevant evidence base, primarily from studies conducted in China (Tang & Xiang, 2021; Tang et al., 2021) and North America (Breen, Lee, & Neimeyer, 2021; Breen, Mancini, et al., 2021; Downar et al., 2022; Neimeyer & Lee, 2021), however findings to date are inconsistent. In particular, there is a lack of consensus on whether bereavement due to COVID-19 deaths is associated with worse outcomes than deaths due to other causes during the pandemic (Breen, Mancini, et al., 2021; Downar et al., 2022; Gang et al., 2022; Shahini et al., 2022); whether and how the context of the death, including physical presence before or at the time of death, is associated with bereavement outcomes (Downar et al., 2022; Neimeyer & Lee, 2021); and whether and how demographic characteristics such as relationship with the deceased relate to bereavement outcomes (Downar et al., 2022; Tang & Xiang, 2021). There has also been little examination of factors associated with bereaved people’s self-reported needs for support.

We conducted a mixed-methods study of bereavement during the pandemic in the UK to contribute to this evidence base. In previous papers, we described sub-optimal end-of-life care, challenging experiences after bereavement, needs for emotional support and barriers to accessing formal and informal support among 711 people bereaved of any cause, using cross-sectional data from an online survey (Harrop et al., 2021; Selman et al., 2022a; Torrens-Burton et al., 2022). In this paper, we determine factors associated with higher levels of grief and bereavement support needs. Our research question was: which pandemic-related challenges and demographic and clinical characteristics are associated with higher levels of grief and support needs among people in the UK bereaved during the COVID-19 pandemic?

## Methods

### Study design

An open web survey (Supplementary file 1) was disseminated to a convenience sample of people bereaved during the pandemic in the UK. The survey was conducted as part of a larger mixed methods study. Study findings regarding experiences of end-of-life care and early bereavement (Harrop, Goss, Longo, et al., 2021; Selman, Farnell, et al., 2022; Torrens-Burton et al., 2022), support use and barriers to formal and informal support (Harrop, Goss, Farnell, et al., 2021), and equity in access to bereavement services from service provider perspectives (L. Selman, Farnell, et al., 2022) have been published separately. This paper builds on this evidence base, particularly our identification of factors associated with poorer experiences of end-of-life care and pandemic-related challenges in early bereavement (Selman, Farnell, et al., 2022), by identifying factors associated with higher levels of grief and support needs. Findings from our earlier analysis included that deaths in hospital/care home increased the likelihood of poorer experiences at the end of life; for example, being unable to visit or say goodbye as wanted (*p* < 0.001). COVID-19 was also associated with worse experiences before and after death; for example, feeling unsupported by healthcare professionals (*p* < 0.001), social isolation/loneliness (OR = 0.439; 95% CI: 0.261–0.739), and limited contact with relatives/friends (OR = 0.465; 95% CI: 0.254–0.852). The deceased being a partner or child increased the likelihood of positive end-of-life care experiences, however being a bereaved partner strongly increased odds of social isolation/loneliness. The current analysis examines how clinical and demographic factors, experiences of end-of-life care and pandemic-related challenges are associated with grief and support needs.

### UK pandemic context

Study participants were bereaved between 16 March 2020, when social-distancing requirements were introduced by the UK Government, and 2 January 2021. During this period, infection control measures and social distancing regulations varied (Institute for Government, 2021). Regulations introduced in March 2020 initially included quarantining for 14 days if symptomatic, avoiding non-essential trips outside the home, limiting all social contact, stopping unnecessary travel, and starting to work from home. This was quickly extended to asking those with serious health conditions to shield from social contact for 12 weeks. On 23 March 2020 the prime minister announced the first lockdown measures ordering people to stay at home; these came onto force legally on 26 March. Schools closed and mass gatherings were banned. On 31 March the Government issued guidance for safe funerals (UK Health Security Agency, 2020) advising social distancing of at least 2 m between attendees, limiting attendees to members of the deceased person’s household or close family members, and people with COVID-19 symptoms not attending. The guidance also strongly advised that mourners should not take part in any rituals or practices that brought them into close contact with the body of a person who had died from or with symptoms of COVID-19. These infection control measures remained in force until June 2020, when restrictions were eased in England, including reopening of pubs, restaurants and hairdressers, followed by indoor theatres and leisure venues from August 2020. Restrictions came into force again from September 2020, with a new three-tier system in place dependent on local infection rates. A second national lockdown was imposed from 5 November to 2 December 2020, followed by an easing of restrictions over Christmas. A third national lockdown started on 6 January 2021.

### Survey development

Survey items and structure were informed by study aims and previous research (Claessen et al., 2013; Harrop, Scott, et al., 2020). The survey was designed with a multi-professional advisory group including social scientists, doctors, psychologists and bereavement counsellors and piloted, refined and tested with 16 bereaved members of the public to ensure acceptability, comprehensiveness and comprehensibility. Non-randomised open and closed questions covered end-of-life and grief experiences, and perceived needs for, access to, and experiences of support.

### Primary outcomes

*Grief* was assessed using the validated 9-item Adult Attitude to Grief (AAG) scale (Sim et al., 2014). The full scale is presented in Table S1 (Supplementary material file 3). This scale was chosen based on the findings of a previous study focused on bereavement outcomes (Harrop, Scott, et al., 2020), which identified the scale as having relatively good ‘fit’ with the ‘coping with grief’ outcome selected and described in this study. The AAG is also widely used by bereavement services in the UK to assess and respond to the needs of bereaved clients (Agnew et al., 2010). The scale is based on the Range of Response to Loss model (Machin et al., 2001), which identifies three distinct responses: being ‘overwhelmed’, a state dominated by emotional/cognitive distress; being ‘controlled’, needing to avoid emotional expression and focus on day-to-day life; and being balanced or ‘resilient’, feeling supported and able to cope. AAG subscale scores indicate levels of feeling overwhelmed, controlled, and reversed resilience on a scale of 0 (none) to 12 (very high). Example items from each respective subscales include: ‘For me, it is difficult to switch off thoughts about the person I have lost’ (strongly agree (4) to strongly disagree (0)); ‘For me, it is important to keep my grief under control’ (strongly agree (4) to strongly disagree (0)); ‘I feel able to face the pain which comes with loss’ (strongly agree (0) to strongly disagree (4)). Internal consistency was acceptable (Cronbach’s α = 0.73, 0.70, and 0.76, respectively). An overall index of vulnerability (IOV) is calculated by summing subscale scores (IOV: 0–20 = low vulnerability, 21–23 = high vulnerability, and 24–36 = severe vulnerability (Sim et al., 2014)).

*Support needs* were assessed in 13 domains, informed by previous studies (Harrop, Morgan, et al., 2020; Harrop, Scott, et al., 2020): Dealing with my feelings about the way my loved one died, Dealing with my feelings about being without my loved one, Expressing my feelings and feeling understood by others, Feeling comforted and reassured, Feelings of anxiety and depression, Loneliness and social isolation, Finding balance between grieving and other areas of life, Regaining sense of purpose and meaning in life, Managing and maintaining my relationships with friends and family, Participating in work, leisure or other regular activities (e.g. shopping, housework), Getting relevant information and advice (e.g. legal, financial, available support), Practical tasks e.g. managing the funeral, registering the death, other paperwork etc., and Looking after myself/family e.g. getting food, medication, childcare. Each domain is assessed on scale from ‘no support needed’ to ‘high level of support needed’. Exploratory factor analysis found two subscales (emotional support; practical support). Cronbach’s α was 0.79, 0.95, and 0.94 for practical support, emotional support, and all items, respectively. Subscale scores are the mean across all subscale items. The overall mean is evaluated over 13 items. We interpret results for both subscale scores and overall mean via: 1 = no support needed; 3 = moderate level needed; 5 = high level needed.

### Associated factors

We assessed whether demographic and clinical factors, experiences of end-of-life care and pandemic-related problems independently predicted levels of grief and support needs. Factors included in the analysis are recognised risk factors for poor bereavement outcomes (age, gender, time since death, relationship to deceased, expectedness of the death, ability to say goodbye to the deceased, experiences of end-of-life care, perceived social support) (Kentish-Barnes et al., 2015; Lobb et al., 2010; Selman et al., 2020; Yamaguchi et al., 2017) or are known to be indirectly associated with such outcomes (qualifications, deprivation level and region; place of death; cause of death) (Haugen et al., 2021; Miyashita et al., 2008). We used postcode data to identify geographical region of residence and (for England) socio-economic deprivation.

### Experiences of end-of-life care

Six items, adapted from the Consumer Quality Index for Palliative Care (Claessen et al., 2013), assessed end-of-life care experiences: involvement in care decisions, knowing contact details for the professional responsible for care, receiving information about the approaching death, support by healthcare professionals immediately after the death, contact by the hospital/care provider after the death, and provision of information about bereavement support services.

### Pandemic-related problems

Six items assessed pandemic-related challenges prior to and after the death, e.g. being unable to visit the person who died prior to their death, restricted funeral arrangements, social isolation and loneliness (see Table S8). All items were answered yes/no. Respondents were asked to tick all experiences that applied to them.

### Study procedure

The survey was administered via Jisc (JISC, 2021), open 28th August 2020 to 5th January 2021 and disseminated via social and mainstream media, voluntary sector associations and bereavement support organisations, including organisations representing ethnic minority communities. Organisations disseminated the survey by sharing on social media, web-pages, newsletters, on-line forums and via direct invitations to potential participants (Supplementary file 2). For ease of access, the survey was posted onto a bespoke study-specific website with a memorable URL (covidbereavement.com). Two participants chose to complete the survey in paper format. Summaries of survey results (including interim results released November 2020 (Harrop E., 2020)) were posted on the website.

Inclusion criteria: aged 18+; family or close friend bereaved since social-distancing requirements were introduced in the UK (16/03/2020); death occurred in the UK; ability to consent. The initial section of the survey requested informed consent and provided data protection information (Supplementary file 1). 12 surveys were completed in duplicate; the first completed survey was retained for these participants. Two incomplete surveys where only the consent question had been answered were excluded.

Reporting follows the Checklist for Reporting Results of Internet E-Surveys (Eysenbach, 2004) (see checklist Supplementary file 4).

### Data analysis

An analysis plan was drafted by a statistician (DJJF) and refined iteratively by the research team. Descriptive statistics were used to describe all variables, with normality of outcomes assessed as appropriate. Standard univariate tests were used first to compare differences between groups for all outcomes. A standardised effect size (Cohen’s *d*) was used to measure differences between groups for ordinal or continuous variables (*d* = 0.3: small effect, *d* = 0.5: medium effect, *d* = 0.8: large effect, *d* = 1.2: very large effect). Where factors contained more than one group, we used the maximum difference in means between any two groups in the factor and the average standard deviation across all groups. By using a standardised measure of effect size, the effects of factors on outcomes could be compared directly and patterns across multiple outcomes ascertained.

IOV scores for the AAG questionnaire were found to be normally distributed via normal plots and appropriate statistical tests of normality (i.e., the Kolgorov-Smirnov test) and so the *t*-test was used for those cases where a factor had two levels (or groups) and one-way ANOVA for all other cases. Note also that often differences are significant at the *p* < 0.001 level, which equates (conservatively) to applying a Bonferroni correction accounting for multiplying testing in all cases. The subscales for the AAG were generally also normally distributed, although the overwhelmed subscale showed some left skew; parametric and non-parametric methods were used in this case (only), although results of both approaches were found to agree strongly (therefore results of only parametric tests are quoted here). In some cases, IOV was classed into low, high, and severe categories to illustrate the results for IOV still further; a simple chi-squared test was then applied to test for this grouped form of IOV with respect to all of the factors. (Results for *p*-values were however found to agree broadly with parametric tests for IOV considered directly). We consider effect sizes directly, in addition to *p*-values, to determine what factors are “important.”

Factors with consistently medium or large effects across multiple outcomes were included in a mixed model of IOV, which complements results of univariate analyses and adjusts for any effects of confounding. A directed acyclic graph (DAG) was also used to map out the relationships between variables, which was useful in planning the analyses and visualising the mixed-effects model. As no readily apparent pattern occurred in IOV with respect to UK region, this was taken to be the random effect; all other factors were taken as fixed effects. IOV reduced with age of the deceased above 40–50 years old and so this was introduced into the mixed model as an explicit (quadratic) covariate. Interactions between variables in the model did not improve the model fit significantly or change regression coefficients greatly. Residuals for this model were normally distributed, as required, and all assumptions of this approach were satisfied. Analysis was conducted in SPSS V26.

### Ethical approval

The study was approved by The University of Bristol School of Medicine Research Ethics Committee (SMREC 20/59) and conducted in accordance with the Declaration of Helsinki. All respondents provided informed consent.

## Results

### Sample characteristics

711 bereaved people participated (Table 1). Participants represented diverse geographical areas, deprivation indexes and levels of education. 88.6% of participants were female (*n* = 628); the mean age of the bereaved person was 49.5 years old (SD = 12.9; range 18–90). The most common relationship of the deceased to the bereaved was parent (*n* = 395, 55.6%), followed by partner/spouse (*n* = 152, 21.4%). 72 people (10.1%) had experienced more than one bereavement. 33 people (4.7%) self-identified as from a minority ethnic background. Missing data was minimal (i.e., close to zero) for all variables and so imputation was not necessary.

**Table 1.** Characteristics of the Bereaved Person.

Age			
	**Mean [Median]**	**SD**	**Min-Max**
Age (years)	49.5 [50.0]	12.9	18–90

	***n***	**Percentage**	
Gender identity			
Male	74	10.4%	
Female	628	88.6%	
Other	7	1.0%	
			
Ethnicity			
			
**Non-Black, Asian or minority ethnic background (total)**	**676**	**95.3%**	
White British	438	64.8%	
White English	111	16.4%	
White Welsh	41	6.1%	
Northern Irish	22	3.3%	
White Scottish	40	5.9%	
Any other white	17	2.5%	
White Irish	7	1.0%	
**Black, Asian or minority ethnic background (total)**	**33**	**4.7%**	
White and Black Caribbean	12	36.4%	
White and Asian	5	15.2%	
Indian	4	12.1%	
Black Caribbean	4	12.1%	
Any other mixed background	3	9.1%	
Pakistani	1	3.0%	
Bangladeshi	1	3.0%	
Arab	1	3.0%	
White and Black African	1	3.0%	
Any other Asian	1	3.0%	

Religious beliefs			
Buddhism	8	1.2%	
Christian	251	36.7%	
Hinduism	3	0.4%	
Islam	5	0.7%	
Judaism	6	0.9%	
Sikhism	2	0.3%	
Other or agnostic	107	15.7%	
No	301	44.1%	
Highest qualification			
None or GCSEs	108	15.3%	
A-level or Apprenticeship or ONC	132	18.6%	
HND or University Degree	468	66.1%	
			
Region			
England	517	78.5%	
Wales	63	9.6%	
Scotland	53	8.0%	
Northern Ireland	26	3.9%	
		
Unemployed during the pandemic?			
Yes	55	7.9%	
No	645	92.1%	
		
Bereavements in previous year?			
Yes	158	22.5%	
No	543	77.5%	
		
IMD Decile (England only) (*n* = 517)			
		
1	26	5.0%	
2	45	8.7%	
3	49	9.5%	
4	52	10.1%	
5	64	12.6%	
6	52	10.1%	
7	58	11.2%	
8	57	11.0%	
9	46	8.9%	
10	50	9.7%	

*Note.* GCSE = General Certificate of Secondary Education for 15 and 16 year olds in the UK; A Levels = Advanced Level subject-based qualification for students in the UK aged 16 and above; ONC = Ordinary National Certificate (equivalent to A Levels); HND = Higher National Diploma (vocational qualification provided by higher or further education colleges in the UK); IMD = indices of multiple deprivation.

Table 2 presents the characteristics of the deceased people. The mean age of the deceased person was 72.2 years old (SD = 16.1; range 4 months gestation to 102 years). 43.8% (*n* = 311) died of confirmed or suspected COVID-19, 21.9% (*n* = 156) from cancer, and 16.7% (*n* = 119) from another life-limiting condition. Most died in hospital (*n* = 410, 57.8%). Questionnaires were completed a median of 152 days (5 months) after the death (range 1–279 days).

**Table 2.** Characteristics of the Deceased Person.

Age			
	**Mean [Median]**	**SD**	**Min-Max**
Age (years)	72.2 [74.0]	16.1	0–102

	***n***	**Percentage**	
Relationship of the deceased person to the Bereaved^a^			
Partner	152	21.4%	
(Male/Female)	(129/23)	(18.1%/3.2%)	
Parent	395	55.6%	
(Father/Mother)	(218/197)	(30.7%/27.7%)	
Grandparent	54	7.6%	
Sibling	23	3.2%	
(Brother/Sister)	(15/10)	(2.1%, 1.4%)	
Child	15	2.1%	
(Son/Daughter)	(12/4)	(1.7%/0.6%)	
Other family member	46	6.5%	
Colleague or friend	26	3.7%	
		
Cause of death			
COVID	273	38.5%	
Suspected COVID	38	5.4%	
Non-COVID (total)	399	56.2%	
Cancer	156	21.9%	
Other PLLC^b^	118	16.7%	
Non-PLLC/SD^c^	112	15.8%	
Don’t know	12	3.0%	
Not specified	1	0.2%	
			
Was the death expected?			
Yes	113	16.0%	
No	552	78.0%	
Don’t know	43	6.1%	
		
Place of death			
In hospital	410	57.7%	
In their home	158	22.2%	
In a hospice	37	5.2%	
In a care home	91	12.8%	
Other/Don’t know	13	1.8%	
		

^a^Multiple bereavements recorded by participants explain discrepancies between overall totals in sibling, child and parent groups and their sub-categories.

^b^PLLC = Progressive life-limiting condition e.g. heart disease, COPD, dementia.

^c^Non-PLLC/SD = Non-progressive life-limiting condition/sudden death e.g. stroke, heart attack, accident, suicide.

## Primary outcomes

### Levels of grief

Individual AAG item and subscale scores are given in Supplementary Tables S1-S2. Mean IOV was 20.41 (95% CI = 20.06 to 20.77, median = 21.00), i.e., demonstrating high levels of vulnerability in grief overall. 48.4% exhibited low levels of vulnerability (i.e., 0 ≤ IOV ≤ 20); 23.4% exhibited high levels (i.e., 21 ≤ IOV ≤ 23), and 28.2% exhibited severe levels (i.e., IOV ≥ 24). Overall subscale scores were: overwhelmed mean = 8.53 (95% CI = 8.31–8.72), controlled mean = 6.61 (95% CI = 6.41–6.82), reversed resilience mean = 5.28 (95% CI = 5.07–5.49).

### Support needs

In 6 (of 13) domains, all relating to psycho-emotional support, 50%–60% of respondents reported high/fairly high levels of need (Table S3). The three most common were: dealing with my feelings about the way my loved one died (60%), expressing my feelings and feeling understood by others (53%), and feelings of anxiety and depression (53%). Subscale scores were: emotional subscale mean = 3.33 (95% CI = 3.25–3.41), i.e., moderate level of emotional support needed; practical subscale, mean = 2.41 (95% CI = 2.34–2.50), i.e., low to moderate level of practical support needed. Overall support (all items) demonstrated a mean = 3.12 (95% CI = 3.04–3.19), i.e., moderate.

## Factors associated with levels of grief and support needs

Effect sizes measured via Cohen’s *d* were used to determine those factors and covariates that had the strongest association with outcomes. We describe significant associations between these factors and the outcomes in detail below, by magnitude of effect (also see Tables 3 and 4 and Supplementary Tables S4-S10).

**Table 3.** Results for Subscales and Scale Scores for the AAG Questionnaire and Support Needed as a Function of the Relationship Between the Bereaved Person and the Deceased.

		AAG				Support Needed		
Person who Died		Overwhelmed	Controlled	Reversed Resilience	IOV	Practical	Emotional	Overall
Partners	*N*	152	148	149	148	150	150	150
	Mean	9.85	6.08	6.00	21.85	2.89	3.76	3.56
	Median	11.00	7.00	6.00	22.00	3.00	3.80	3.62
	SD	2.43	2.78	2.86	4.13	1.14	0.92	0.87
Parents	*N*	393	392	391	390	387	387	387
	Mean	8.49	6.63	5.14	20.26	2.40	3.31	3.10
	Median	9	7	5	20	2.33	3.4	3.15
	SD	2.72	2.63	2.78	4.98	1.02	1.05	0.95
Grandparents	*N*	54	54	54	54	50	52	52
	Mean	7.02	7.44	5.05	19.5	1.81	2.90	2.66
	Median	7	7.75	5	20	1.33	2.9	2.65
	SD	2.70	2.57	2.93	4.17	0.95	1.12	1.01
Sibling	*N*	22	22	23	22	22	23	23
	Mean	8.64	6.77	5.48	21.14	2.08	3.3652	3.07
	Median	9	7.5	5	21.5	1.67	3.63	3.31
	SD	2.52	2.93	2.794	3.81	1.14	1.17	1.07
Child	*N*	15	15	15	15	15	15	15
	Mean	10.13	5.93	6.53	22.6	3.24	3.84	3.71
	Median	11	6	6	22	3.33	4	3.92
	SD	2.59	2.84	2.59	2.64	0.99	0.98	0.93
Other family member	*N*	45	45	45	45	43	45	45
	Mean	6.72	7.24	4.22	18.19	1.90	2.67	2.51
	Median	7	7	4	19	1.67	2.6	2.38
	SD	2.3	3.07	2.81	5.04	1.03	1.14	1.03
Colleague or friend	*N*	24	24	24	24	23	24	24
	Mean	6.58	6.96	4.67	18.21	1.67	2.84	2.59
	Median	7	7	5	18	1.33	2.8	2.5
	SD	2.83	2.29	1.95	4.65	0.87	1.15	1.01
Maximum Cohen’s |*d*|		1.37	0.55	0.86	1.05	1.54	1.09	1.22
One-way ANOVA: *p* =		<0.001	0.032	0.001	<0.001	<0.001	<0.001	<0.001

**Table 4.** Results for Subscales and Scale Scores for the AAG Questionnaire and Support Needed for Social Isolation and Loneliness (No/Yes).

		AAG				Support Needed		
		Overwhelmed	Controlled	Reversed Resilience	IOV	Practical	Emotional	Overall
No	*N*	233	230	231	229	227	228	228
	Mean	7.62	6.87	4.49	18.99	2.07	2.74	2.59
	Median	8	7	4	19	2	2.65	2.46
	SD	2.89	2.6	2.64	5.07	0.99	1.10	0.99
Yes	*N*	472	470	470	469	463	468	468
	Mean	8.98	6.49	5.67	21.11	2.58	3.62	3.39
	Median	9	7	5	21	2.67	3.70	3.46
	SD	2.634	2.757	2.821	4.463	1.11	0.95	0.89
Cohen’s |*d*|		0.49	0.14	0.43	0.44	0.49	0.87	0.85
*t*-test: *p* =		<0.001	0.08	<0.001	<0.001	<0.001	<0.001	<0.001

### Relationship between the bereaved and the deceased

Strong differences (one-way ANOVA: *p* < 0.001) occur for all AAG subscales and IOV and practical, emotional, and overall support needs as a function of the relationship of the deceased person to the bereaved. In particular, IOV, emotional and overall support need scores were much higher for close family, particularly when the person who died was a child or partner (followed by sibling or parent), compared with more distant family members and colleagues or friends (Table 3, Table S4). In the mixed model, relationship of the deceased person to the bereaved showed strong differences in IOV, with close relationships having significantly (one-way ANOVA: *p* = 0.002) higher IOV than more distant relationships.

### Age of the deceased

**Figure 1.**
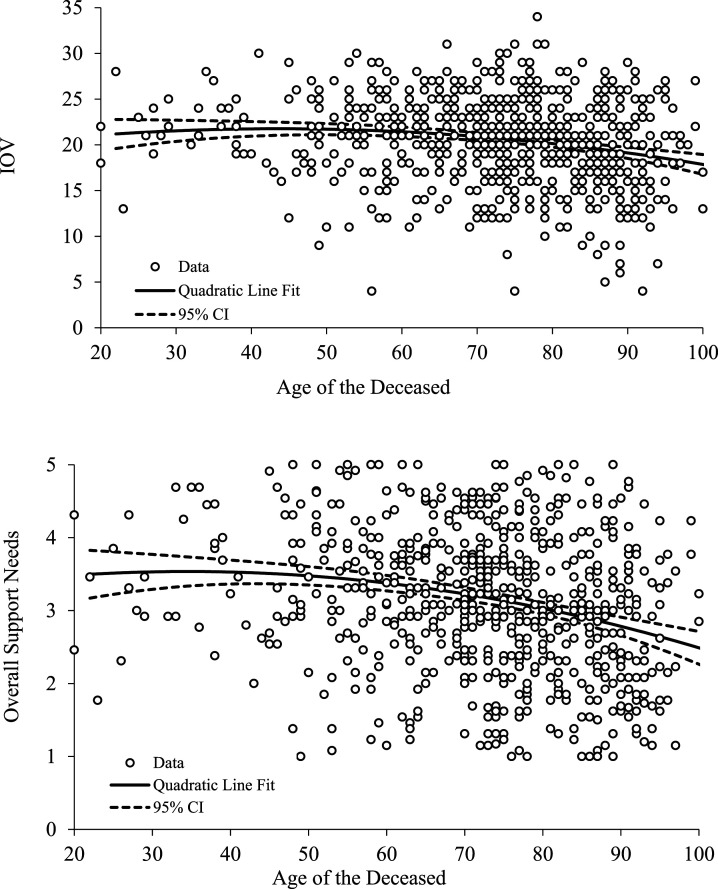
Scatter plots showing levels of grief (IOV) and overall support score as a function of the age of the person who died. Quadratic line fits and associated 95% confidence intervals on the estimate have been added to these figures to show the general trend more clearly, namely, of a distinct reduction in these outcomes with age above 40–50 years.

The age of the deceased person appeared to have a distinct association with levels of grief and perceived support needs; in particular, there were distinct trends of reductions in most of these outcomes for ages above 40–50 years old (Figure 1). This negative trend in IOV with age was also apparent in the mixed model (not shown here).

### Social isolation and loneliness

Bereaved participants who experienced social isolation and loneliness experienced significantly higher (t-test: *p* < 0.001) levels of grief and needed more support (especially emotional support, where a large effect size was observed) than those who did not (Table 4). Overall, 58% of those who experienced social isolation/loneliness reported high or severe levels of vulnerability as measured by IOV, compared with 38.4% of those who did not. The absolute measure of effect size was 19.6% (Cohen’s *h* = 0.46).

### Support from health professionals immediately after the death

Increased levels of perceived support from health professionals were associated with significantly (one-way ANOVA: *p* < 0.001) lower levels of grief measured by IOV, the reversed resilience subscale of the AAG, and emotional and overall support need (small to medium effect, *p* < 0.001) (Table S5). This is also seen in the mixed model, where a distinct and highly significant (*p* < 0.001) decrease in IOV is seen with increasing levels of healthcare professional support immediately after the death. Other aspects of end-of-life experiences were less strongly associated with outcomes and these associations were not significant.

### Place of death

Place of death was significantly associated with all outcomes, with values for the subscale and overall scores for the AAG and support needed lower when the person died “in a care home” compared with the other groups (often *p* < 0.05 from one-way ANOVA), particularly for emotional and overall support, IOV and the overwhelmed AAG subscale (Table S6). A trend of reduced IOV for deaths in care homes (*p* = 0.056) compared with other places where the death occurred was also seen in the mixed model.

### Qualifications

Highest qualification was significantly associated (one-way ANOVA: *p* = 0.019) with level of grief, with more highly educated participants (post A-level qualifications) having slightly lower IOV compared with participants with lower levels of qualification (Table S7). This was maintained in the mixed model (*p* = 0.019).

### Funeral restrictions

Restricted funeral arrangements also had a strong negative association with grief assessed via IOV (Table S8). Experiencing other pandemic-related problems such as being unable to visit, spend time with or say goodbye to a friend or relative prior to their death had a generally small effect (and often *p* > 0.05 via t-tests) on levels of grief and perceived support needs (Table S8).

### Other factors

In univariate analyses, cause of death (COVID vs. non-COVID) had a small but often significant (t-test: *p* < 0.01) effect on levels of grief and perceived support needs (emotional, practical and overall), which were slightly higher for COVID deaths compared to non-COVID (Table S9). However, differences in IOV by cause of death were not significant (*t*-test: *p* = 0.66) in the mixed model. Those who did not expect their loved one to die demonstrated higher levels of grief and also support needs (again often: *p* < 0.001 via t-tests) (Table S10), although this was not significant in the mixed model (*p* = 0.089).

For age of the bereaved person, there was a distinct dip in the scatterplots, with levels of grief and support needs falling up to age approximately 50 and then rising (Supplementary Figure S1). Deprivation, gender, ethnicity and length of time since the death had small effects on outcomes and any observed differences were not statistically significant (one-way ANOVA: *p* > 0.05) (data not shown).

## Discussion

In this national survey of people bereaved during the first 9 months of the pandemic in the UK, we found that relationship to the deceased was the factor most strongly associated with both higher vulnerability in grief and higher support needs. Bereaved people who had lost a partner, child or sibling showed higher levels of grief and support needs compared with bereavements of more distant relatives/friends. Age of the deceased had a strong effect, with younger age associated with both higher vulnerability in grief and higher support needs. Social isolation and loneliness had a medium-large effect on vulnerability in grief and support needs, particularly on emotional support needs. Other factors associated with poorer outcomes were less perceived support from health professionals after the death (with a small-medium effect on vulnerability in grief and on emotional and overall support), place of death being in hospital, hospice or at home rather than in a care home (small-medium effect on vulnerability in grief and on emotional and overall support), and, for vulnerability in grief only, lower level of qualification and experiencing funeral restrictions.

The association between relationship to the deceased and higher levels of grief and support needs coheres with pre-pandemic studies (Aoun et al., 2015; Lobb et al., 2010) and some studies of pandemic bereavement (Breen, Lee, & Neimeyer, 2021;Chen & Tang, 2021; Tang & Xiang, 2021; Tang et al., 2021). A US survey of people bereaved by COVID-19 (*n* = 307) found a close relationship with the deceased (partner or immediate family) was associated with higher functional impairment than a more distant relationship (extended family, friend/acquaintance) (Breen, Lee, & Neimeyer, 2021). Similar findings have been reported in China (Tang & Xiang, 2021), but not in Canada (Downar et al., 2022). We found the deceased being younger was associated with worse grief outcomes and experiences, which echoes other studies (Aoun et al., 2015;Chen & Tang, 2021; Ringdal et al., 2001).

Our findings support research demonstrating that social isolation and loneliness are associated with worse bereavement outcomes (Chen, 2022; Harrop, Scott, et al., 2020; Mason et al., 2020; Scott et al., 2020) and highlight the challenges of bereavement during a time of huge disruptions to social networks. In our sample, as reported previously (Selman, Farnell, et al., 2022), 66.7% reported experiencing social isolation and loneliness when their loved one died, with the odds of social isolation and loneliness highest for bereaved partners compared with other relationships to the deceased, and in COVID-19 deaths compared with other causes of death. The current analysis found reporting social isolation and loneliness was associated with higher vulnerability in grief and higher support needs overall. In a US study of adolescents, adults and healthcare workers in 2020, loneliness was the most common predictor of clinically significant psychiatric symptoms including depression, anxiety, and posttraumatic stress disorder (PTSD) symptoms, suicidal ideation or behaviour, and grief reactions (Murata et al., 2021).

Our qualitative findings (published separately (Torrens-Burton et al., 2022)) demonstrate how lockdown restrictions and shielding prevented access to the mutual comfort and support bereaved people needed to process their grief, explicating the mechanisms at play. Taken together, this evidence highlights the need to support people at risk of social isolation and consider underlying factors and sequelae.

The finding that perceived support from healthcare professionals after death affects levels of grief and support needs demonstrates the importance of compassionate care, timely communication and support around the time of death. This supports findings of pre-pandemic studies (Kentish-Barnes et al., 2015; Yamaguchi et al., 2017), and suggests that poor end-of-life care experiences during the pandemic (Mayland et al., 2021; Neimeyer & Lee, 2021) will lead to higher levels of support needs and demand on bereavement services. Similarly, our finding that lower levels of education are associated with poorer outcomes reflects the findings of previous research (Mason et al., 2020; Milic et al., 2017), underlining the importance of considering structural disadvantage and inequity in bereavement support (Bindley et al., 2019). We found that outcomes were slightly better when a death had occurred in a care home compared with other settings, which may be due to anticipatory grief work, e.g. in the context of dementia diagnoses. This hypothesis is supported by US research that found that deaths from dementia during the pandemic were negatively associated with probable PGD compared with deaths from other causes (Gang et al., 2022).

Pre-pandemic research regarding the impact of funerals on bereavement outcomes is inconclusive (Burrell & Selman, 2020), however even though only 7% of our sample reported not experiencing funeral restrictions, analysis found funeral restrictions were nevertheless associated with poorer outcomes. The impact of funeral restrictions and social isolation in the context of pandemic lockdowns, social distancing and quarantining was also reflected in our qualitative data (Torrens-Burton et al., 2022). Similarly, participants in a survey by Mitima-Verloop et al. rated the impact of the pandemic on their experience of both the funeral service and post-funeral grief rituals as very negative (Mitima-Verloop et al., 2022). However, this did not translate into any significant differences in funeral attendance, funeral evaluation, and the performance and helpfulness of individual and collective rituals between participants bereaved before (*n* = 50) versus during (*n* = 182) the pandemic (Mitima-Verloop et al., 2022), suggesting a complex picture worthy of additional exploration.

Contrary to a US study (Neimeyer & Lee, 2021), we found pandemic-related problematic experiences such as being unable to visit or say goodbye prior to a relative/friend’s death were not significantly associated with levels of grief or support needs. In contrast, participants gave detailed qualitative descriptions of the negative impacts that clinical and social restrictions have had on their grief, e.g. feelings of intense sadness, guilt or anger (Torrens-Burton et al., 2022). These findings help explain why 60% of the sample reported high/fairly high needs for help dealing with how their loved one died. It is possible the impact of these experiences is not fully assessed by the AAG, which provides a broad profile of both core bereavement reactions and the coping response to them and thus an overview of a person’s vulnerability and resilience (Sim et al., 2014). In contrast to other grief measures, including measures of PGD (Boelen & Smid, 2017), the AAG focuses on responses to grief, rather than identifying specific symptoms, such as longing for the dead person, guilt, anger, and impact on identity and functioning, some of which may be particularly relevant in the pandemic context (L.E. Selman et al., 2021). PGD will be assessed and reported at later timepoints in this ongoing longitudinal study, as it should be assessed ≥6 months after a death.

A median of 5 months post-death, we found high levels of vulnerability in grief overall, with 23.4% exhibiting high levels of vulnerability via the AAG (21 ≤ IOV ≤ 23), and 28.2% severe levels of vulnerability (IOV ≥ 24). Public health models of bereavement (Aoun et al., 2015; Aoun et al., 2012) suggest that in non-pandemic times, 10% of bereaved people are at high risk of complex grief issues and may need professional mental health support, and a further 30% are at moderate risk and may need some additional support e.g. via peer support groups. Since acute grief is one of the strongest predictors of future disturbed grief (Boelen et al., 2019; Milic et al., 2017), and AAG scores correlate with measures of PGD (Sim et al., 2014), our findings support the hypothesis that grief disorder prevalence will rise because of the pandemic (Maarten C. Eisma & Boelen, 2021).

Some US research suggests that bereavement due to COVID-19 might be associated with elevated acute grief and post-traumatic stress, depression, and anxiety symptoms compared with non-COVID-19 bereavement (Breen, Lee, & Neimeyer, 2021). In China, a survey of COVID-19 bereaved adults, including a subset bereaved >6 months ago, found elevated posttraumatic stress, anxiety, and depression symptoms, with 38%–29% meeting criteria for PGD (Tang & Xiang, 2021; Tang et al., 2021). Lee et al. studied grief experiences among adult mourners who lost a loved one to COVID-19 and found universal endorsement of one or more forms of self-blame (guilt, regret, shame) or unfinished business, with over one-third of mourners endorsing all four experiences (Lee et al., 2022). In our current analysis, COVID-19 was associated with slightly higher severity of grief and support needs in univariate calculations, although this was not significant in the mixed model for IOV. In previous analyses we found that compared with non-COVID bereavement, the COVID-19 bereaved had worse experiences of end-of-life and early bereavement, including higher rates of social isolation and loneliness (Selman, Farnell, et al., 2022). This is contrary to two comparative US studies by Eisma et al. which found that social support was not experienced differently across loss types, although both these studies are limited by small convenience samples of COVID-19 bereaved (*n* = 99 and *n*-49 respectively) (Maarten C. Eisma & Tamminga, 2022; Maarten C. Eisma et al., 2021). Further research is needed to help explicate relevant mechanisms and impact.

The higher levels of grief observed in this sample compared with pre-pandemic studies may be due to stressors universally experienced during the pandemic rather than specific to COVID-19 bereavements (Eisma & Tamminga, 2020), including disruptions to meaning-making processes following a death (Breen, Mancini, et al., 2021), and the overall mental health impact of living through the pandemic (Joaquim et al., 2021; Kira et al., 2021). Eisma et al. (Eisma & Tamminga, 2020) found that people in the Netherlands who recently experienced a non-COVID-19 death during the pandemic reported higher levels of acute grief than those recently bereaved before the pandemic. Breen et al. (Breen, Mancini, et al., 2021) found that among people bereaved during the pandemic in the US (*N* = 409), there were no significant differences in grief outcomes or functional impairment according to cause of death. Further research is required to establish which experiences and symptomatology are specific to the COVID-19 bereaved and which apply to the broader population of bereaved people.

### Strengths and weaknesses

The sample was large, with good spread across geographical areas, education and deprivation, but relied on voluntary response sampling and was biased towards female and white respondents, despite targeted advertising to men and people from minoritised ethnic communities. By recruiting mostly online, we were less likely to reach the very old or other digitally marginalised groups. Through subsequent qualitative interviews, we have explored in depth the experiences of people from communities and groups less well represented in the survey (publication forthcoming). Convenience sampling might have resulted in more people with negative experiences participating. Despite these limitations, group sizes were sufficient to enable comparisons (although not to the level of specific ethnic groups) and, while not providing population-level prevalence data, the sample does enable identification of potential risk factors to inform future practice and policy.

### Implications for research

Longitudinal population-based studies are needed to establish how pandemic bereavement might affect health outcomes, including rates of PGD, and needs for informal and formal bereavement support. In-depth research exploring the needs of bereaved people from minoritised ethnic backgrounds, same-sex couple, men, children and young people, and people with pre-existing mental health conditions (Joaquim et al., 2021) is also required. The current study includes follow-up surveys at c.7, 13 and 25 months post-death and qualitative interviews which will add to our understanding of pandemic bereavement.

## Conclusions and implications for policy and practice

Study findings highlight the complexities and challenges of pandemic bereavement as well as identifying who is potentially at risk of poor bereavement outcomes and higher levels of support need. We make the following recommendations to inform bereavement support and policy in this and future pandemics:

1. Given high levels of grief vulnerability and needs for bereavement support, especially psycho-emotional support, among people bereaved during the pandemic, statutory, voluntary and community bereavement support services require increased investment, underpinned by national and local policies.
2. In addition, following public health strategies, compassionate community-based initiatives in bereavement are needed to strengthen, support and learn from communities’ own approaches to informal bereavement support.
3. Close relatives and people less able to advocate for themselves may be at particular risk of poor outcomes, especially when socially isolated, and should be targeted for additional support and follow-up.
4. The importance of funerals and other group mourning social practices must be recognised, with restrictions considered carefully. Funeral providers and celebrants play an important role in providing alternative meaningful services in the contexts of restrictions (Burrell & Selman, 2020; Mitima-Verloop et al., 2022).
5. The quality of care and support provided to bereaved people immediately after a death is associated with bereavement outcomes and must be prioritised and adequately resourced across care settings in the pandemic context, just as in non-pandemic times.

## Supplemental Material

Supplemental Material - Factors Associated With Higher Levels of Grief and Support Needs Among People Bereaved During the Pandemic: Results from a National Online Survey Supplemental Material for Factors Associated With Higher Levels of Grief and Support Needs Among People Bereaved During the Pandemic: Results from a National Online Survey by Lucy E. Selman, Damian J. J. Farnell, Mirella Longo, Silvia Goss, Anna Torrens-Burton, Kathy Seddon, Catriona R. Mayland, Linda Machin, Anthony Byrne, and Emily J. Harrop in OMEGA - Journal of Death and Dying

Supplemental Material - Factors Associated With Higher Levels of Grief and Support Needs Among People Bereaved During the Pandemic: Results from a National Online Survey Supplemental Material for Factors Associated With Higher Levels of Grief and Support Needs Among People Bereaved During the Pandemic: Results from a National Online Survey by Lucy E. Selman, Damian J. J. Farnell, Mirella Longo, Silvia Goss, Anna Torrens-Burton, Kathy Seddon, Catriona R. Mayland, Linda Machin, Anthony Byrne, and Emily J. Harrop in OMEGA - Journal of Death and Dying
