# Supplementary material for: Factors Associated With Higher Levels of Grief and Support Needs Among People Bereaved During the Pandemic: Results from a National Online Survey
Source: Omega (Westport). 2022 Dec 21;91(2):904–31. doi: 10.1177/00302228221144925 (PMC12018716; doi:10.1177/00302228221144925)

Supplementary Tables and Figures

S1: Frequency table for items in the AAG questionnaire (n = 711)

AAG item (and score)		4 = Strongly agree n (%)	3 = Agree n (%)	2= Neither agree or disagree n (%)	1= Disagree n (%)	0 = Strongly disagree n (%)	Missing n (%)	Mean (95% CI)	Median
Overwhelmed items	2. For me, it is difficult to switch off thoughts about the person I have lost.	309 (43.5)	217 (30.5)	95 (13.4)	58 (8.2)	27 (3.8)	5 (0.7)	3.02 (2.93 to 3.1)	3
	5. I feel that I will always carry the pain of grief with me.	330 (46.4)	230 (32.3)	79 (11.1)	47 (6.6)	18 (2.5)	7 (1)	3.15 (3.07 to 3.23)	3
	7. Life has less meaning for me after this loss.	174 (24.5)	172 (24.2)	157 (22.1)	129 (18.1)	69 (9.7)	10 (1.4)	2.36 (2.26 to 2.45)	2
Controlled items	4. I believe that I must be brave in the face of loss.	102 (14.3)	266 (37.4)	151 (21.2)	126 (17.7)	57 (8)	9 (1.3)	2.33 (2.24 to 2.42)	3
	6. For me, it is important to keep my grief under control.	93 (13.1)	284 (39.9)	164 (23.1)	107 (15)	52 (7.3)	11 (1.5)	2.37 (2.28 to 2.45)	3
	8. I think it's best just to get on with life in spite of this loss.	44 (6.2)	200 (28.1)	203 (28.6)	153 (21.5)	97 (13.6)	14 (2)	1.92 (1.82 to 1.99)	2
**AAG item (and score)**		**0 = Strongly agree n (%)**	**1 = Agree n (%)**	**2 = Neither agree or disagree n (%)**	**3 = Disagree n (%)**	**4 Strongly disagree n (%)**	**Missing n (%)**	**Mean (95% CI)**	**Median**
Reversed Resilience Items	1. I feel able to face the pain which comes with loss.	55 (7.7)	228 (32.1)	126 (17.7)	173 (24.3)	116 (16.3)	13 (1.8)	2.1 (2.00 to 2.19)	2
	3. I feel very aware of my inner strength when faced with grief.	88 (12.4)	225 (31.6)	202 (28.4)	141 (19.8)	45 (6.3)	10 (1.4)	1.76 (1.67 to 1.84)	2
	9. It may not always feel like it but I do believe that I will come through this experience of grief.	111 (15.6)	333 (46.8)	145 (20.4)	73 (10.3)	39 (5.5)	10 (1.4)	1.42 (1.35 to 1.51)	1

Table S2: Descriptive statistics for the AAG questionnaire

Subscales/ Total score	*n*	% Missing	Mean (95% CI) [Median]	SD
Overwhelmed	705	0.8%	8.53 (8.31 to 8.72) [9.00]	2.79
Controlled	700	1.5%	6.61 (6.41 to 6.82) [7.00]	2.71
Reversed Resilience	701	1.4%	5.28 (5.07 to 5.49) [5.00]	2.82
IOV	698	1.8%	20.41 (20.06 to 20.77) [21.00]	4.77
IOV Categories	*n (%)*			
Low (IOV = 0-20)	338 (48.4)			
High (IOV = 21-23)	163 (23.4)			
Severe (IOV = 24-36)	197 (28.2)			

Table S3: Support needs ranked by mean level of need

	High or fairly high level of support needed	Moderate level of support needed	Little or no support needed	Mean (95% CI)	Median
Dealing with my feelings about the way my loved one died	59.8%	21.5%	18.7%	3.71 (3.62 to 3.80)	4
Dealing with my feelings about being without my loved one	49.9%	29.3%	20.8%	3.48 (3.39 to 3.57)	3
Expressing my feelings and feeling understood by others	53%	23.9%	23%	3.48 (3.38 to 3.57)	4
Feeling comforted and reassured	51.8%	26.7%	21.6%	3.46 (3.37 to 3.55)	4
Feelings of anxiety and depression	52.8%	21.1%	26.1%	3.45 (3.35 to 3.55)	4
Loneliness and social isolation	52.0%	19.1%	29%	3.36 (3.26 to 3.46)	4
Finding balance between grieving and other areas of life	45.0%	27.9%	27%	3.29 (3.20 to 3.39)	3
Regaining sense of purpose and meaning in life	46.7%	21.6%	31.7%	3.26 (3.15 to 3.36)	3
Managing and maintaining my relationships with friends and family	36.2%	26.4%	37.4%	2.98 (2.88 to 3.08)	3
Participating in work, leisure or other regular activities (e.g. shopping, housework)	33.8%	23.9%	42.1%	2.87 (2.76 to 2.97)	3
Getting relevant information and advice e.g. legal, financial, available support	24.3%	22.3%	53.3%	2.51 (2.41 to 2.61)	2
Practical tasks e.g. managing the funeral, registering the death, other paperwork etc.	23.5%	21.7%	54.7%	2.48 (2.38 to 2.58)	2
Looking after myself/family e.g. getting food, medication, childcare	15.2%	22.8%	62%	2.25 (2.16 to 2.34)	2

Note to interpret means and medians: no support = 1; little support = 2; moderate support = 3; fairly high support = 4; and high support = 5

Table S4: Results for the three IOV groups (i.e., low, high, & extreme) by relationship of bereaved person to the deceased. (Overall association between IOV and relationship: Chi-squared test: *P* < 0.001; "row" percentages shown are shown below, i.e., with respect to *n* quoted in each row for each type of relationship to the deceased.)

	n	Low	High	Severe
Partners	148	34.5%	28.4%	37.2%
Parents	390	50.3%	21.0%	28.7%
Grandparents	54	57.4%	27.8%	14.8%
Sibling	22	36.4%	40.9%	22.7%
Child	15	20.0%	40.0%	40.0%
Other family member	45	68.9%	15.6%	15.6%
Colleague or friend	24	75.0%	8.3%	16.7%

Table S5: Results for subscales and overall scale scores for the AAG questionnaire and support needed as a function of "Did you feel well supported by the healthcare professionals immediately after the death of your loved one?"

		AAG				Support Needed		
		Overwhelmed	Controlled	Reversed Resilience	IOV	Practical	Emotional	Overall
Very well supported	*n*	95	95	95	95	93	93	93
	Mean	8.21	6.06	4.42	18.69	2.40	3.06	2.90
	SD	2.58	2.74	2.66	4.50	1.00	1.04	0.96
	Median	8	6	4	20	2.33	3.1	2.92
Fairly well supported	*N*	105	103	103	103	105	104	104
	Mean	8.72	6.93	5.14	20.73	2.43	3.31	3.10
	SD	2.88	2.72	2.56	4.78	1.06	1.05	0.96
	Median	9	7	5	21	2.33	3.35	3.08
A little bit supported	*n*	138	137	139	137	136	136	136
	Mean	8.77	6.63	5.1	20.46	2.54	3.39	3.20
	SD	2.60	2.54	2.71	4.55	1.07	1.02	0.94
	Median	9	7	5	21	2.5	3.55	3.23
Not at all supported	*n*	250	248	247	246	251	249	249
	Mean	9.11	6.41	5.93	21.46	2.61	3.59	3.36
	SD	2.71	2.77	2.95	4.62	1.12	1.05	0.96
	Median	10	7	6	22	2.33	3.7	3.38
Maximum Cohen's $\|d\|$		0.33	0.32	0.56	0.60	0.20	0.50	0.47
One-way ANOVA: P =		0.05	0.13	<0.001	<0.001	0.28	<0.001	0.001

Table S6: Results for subscales and scale scores for the AAG questionnaire and support needed as a function of place of death

		AAG				Support Needed		
		Overwhelmed	Controlled	Reversed Resilience	IOV	Practical	Emotional	Overall
In hospital	*n*	406	404	406	403	395	400	400
	Mean	8.84	6.45	5.52	20.79	2.51	3.44	3.23
	SD	2.76	2.76	2.92	4.64	1.14	1.05	0.97
	Median	9	7	5	21	2.33	3.55	3.23
In their home	*N*	157	156	156	156	152	154	154
	Mean	8.31	7.01	5.04	20.34	2.40	3.24	3.05
	SD	2.74	2.50	2.81	4.85	1.10	1.15	1.06
	Median	9	7	5	21	2.33	3.2	3
In a hospice	*n*	37	35	35	35	37	37	37
	Mean	8.86	6.94	5.20	20.83	2.24	3.36	3.10
	SD	2.69	2.86	2.56	5.46	0.95	0.89	0.81
	Median	8	8	5	21	2	3.3	3
In a care home	*n*	90	90	89	89	91	90	90
	Mean	7.34	6.68	4.65	18.70	2.08	2.96	2.75
	SD	2.85	2.67	2.35	4.78	0.88	1.06	0.96
	Median	7.5	7	4	19	2	3	2.77
Other / Do not Know	*n*	13	13	13	13	13	13	13
	Mean	9.08	5.31	5.08	19.46	2.51	3.57	3.32
	SD	2.47	3.04	2.75	3.80	1.16	1.37	1.22
	Median	9	6	5	19	2.33	4	3.62
Cohen's *d*		0.64	0.61	0.32	0.45	0.41	0.55	0.57
Maximum Cohen's |*d*|		<0.001	0.10	0.05	0.01	0.01	0.01	0.003
One-way ANOVA: *P* =		<0.001	0.158	0.11	0.002	0.036	0.002	0.001

Table S7: Results for subscales and scale scores for the AAG questionnaire and support needed as a function of highest qualification.

		AAG				Support Needed		
		Overwhelmed	Controlled	Reversed Resilience	IOV	Practical	Emotional	Overall
None / GCSEs	*n*	103	101	102	100	104	104	104
	Mean	9.48	5.94	6.42	21.77	2.63	3.52	3.32
	SD	3.00	2.83	2.94	4.1	1.19	1.07	1.02
	Median	10	6	6.5	22	2.67	3.68	3.40
A-level/ apprenticeship / ONC	*n*	132	132	131	131	127	129	129
	Mean	9.29	6.61	5.55	21.48	2.53	3.57	3.33
	SD	2.56	2.67	2.84	4.52	1.08	1.06	0.98
	Median	10	7	6	22	2.33	3.8	3.38
HND / University Degree / Postgraduate (etc)	*n*	467	464	465	464	456	460	460
	Mean	8.11	6.76	4.94	19.8	2.32	3.22	3.02
	SD	2.71	2.68	2.70	4.86	1.06	1.07	0.98
	Median	8	7	5	20	2	3.25	3
Maximum Cohen's $\|d\|$		0.50	0.30	0.52	0.44	0.28	0.33	0.32
One-way ANOVA: P =		<0.001	0.03	<0.001	<0.001	0.02	0.001	0.001

Table S8: Percentages for the 3 groups with respect to IOV (low, high, severe) for pandemic-related problems.

		n =	IOV Group			Cohen's *h*	*P* =
			Low	High	Severe		
Unable to visit them prior to their death	No	320	49.7%	24.4%	25.9%	0.088	0.459
	Yes	378	47.4%	22.5%	30.2%		
Limited contact with them in last days of their life	No	293	47.1%	25.3%	27.6%	0.068	0.596
	Yes	405	49.4%	22.0%	28.6%		
Unable to say goodbye as I would have liked	No	253	49.8%	23.3%	26.9%	0.050	0.811
	Yes	445	47.6%	23.4%	29.0%		
Restricted funeral arrangements[†]	No	45	66.7%	8.9%	24.4%	0.477	0.018
	Yes	653	47.2%	24.3%	28.5%		
Social isolation and loneliness	No	229	61.6%	19.7%	18.8%	0.460	<0.001
	Yes	469	42.0%	25.2%	32.8%		
Limited contact with other close relatives or friends	No	131	48.9%	23.7%	27.5%	0.019	0.994
	Yes	567	48.3%	23.3%	28.4%		

Note: The same sizes for the responses "no" and "yes" are shown for each question. Results for Cohen's *h* measure of effect size between two proportions indicates zero to small effect sizes only. *P*-values are from chi-squared analysis. "Row" percentages shown are shown below, i.e., with respect to *n* quoted in each row for yes / no answers for each type of pandemic related problem.

[†]Sample sizes are a little low here for some groups (low, high, severe) for "no" to "restricted funeral arrangements", which is possibly why $P = 0.018$ (rather than, say, $P < 0.001$ as for social isolation and loneliness).

Table S9: Results for subscales and scale scores for the AAG questionnaire and support needed as a function of cause of death, i.e., COVID-19 (confirmed or suspected) or non-COVID-19

		AAG				Support Needed		
		Overwhelmed	Controlled	Reversed Resilience	IOV	Practical	Emotional	Overall
COVID	*n*	306	305	305	303	302	304	304
	Mean	8.95	6.40	5.70	21.07	2.53	3.55	3.32
	SD	2.73	2.84	2.96	4.49	1.09	0.98	0.92
	Q_1	7	4.5	3	18	1.67	3	2.79
	Q_2 (Median)	9	6	5	22	2.33	3.65	3.31
	Q_3	11	8	8	24	3.33	4.3	4
Non-Covid	*n*	398	394	395	394	387	391	391
	Mean	8.21	6.78	4.97	19.91	2.32	3.17	2.97
	SD	2.81	2.6	2.66	4.93	1.09	1.13	1.03
	Q_1	6	5	3	17	1.33	2.30	2.23
	Q_2 (Median)	8	7	5	20	2	3.2	3
	Q_3	11	9	7	23	3	4	3.69
Cohen's $\|d\|$		0.27	0.14	0.26	0.25	0.19	0.36	0.35
t-test: P =		<0.001	0.068	0.001	0.001	0.012	<0.001	<0.001

Table S10: Results for subscales and scale scores for the AAG questionnaire and support needed as a function of the question "Did you expect your loved one to die around this time? (e.g. if they had a terminal illness)"

		AAG				Support Needed		
		Overwhelmed	Controlled	Reversed Resilience	IOV	Practical	Emotional	Overall
Yes	*n*	113	113	113	113	108	110	110
	Mean	7.56	6.91	4.35	18.81	2.15	2.98	2.787
	SD	2.75	2.31	2.58	4.83	1.01	1.03	0.96
	Median	8	7	4	19	2	3	2.82
No	*n*	547	543	543	541	538	541	541
	Mean	8.83	6.5	5.56	20.88	2.49	3.45	3.23
	SD	2.74	2.79	2.84	4.72	1.11	1.07	0.99
	Median	9	7	5	21	2.33	3.6	3.25
Cohen's $\|d\|$		0.47	0.36	0.46	0.46	0.33	0.58	0.57
P =		<0.001	0.032	<0.001	<0.001	0.002	<0.001	<0.001

Figure S1: Scatter plots showing the IOV and overall support score as a function of the age of the bereaved person. Quadratic line fits with associated 95% confidence intervals of the estimate have been added to these figures also in order to show the general trend of a minimum in the outcomes at about an age of 50 years old more clearly.

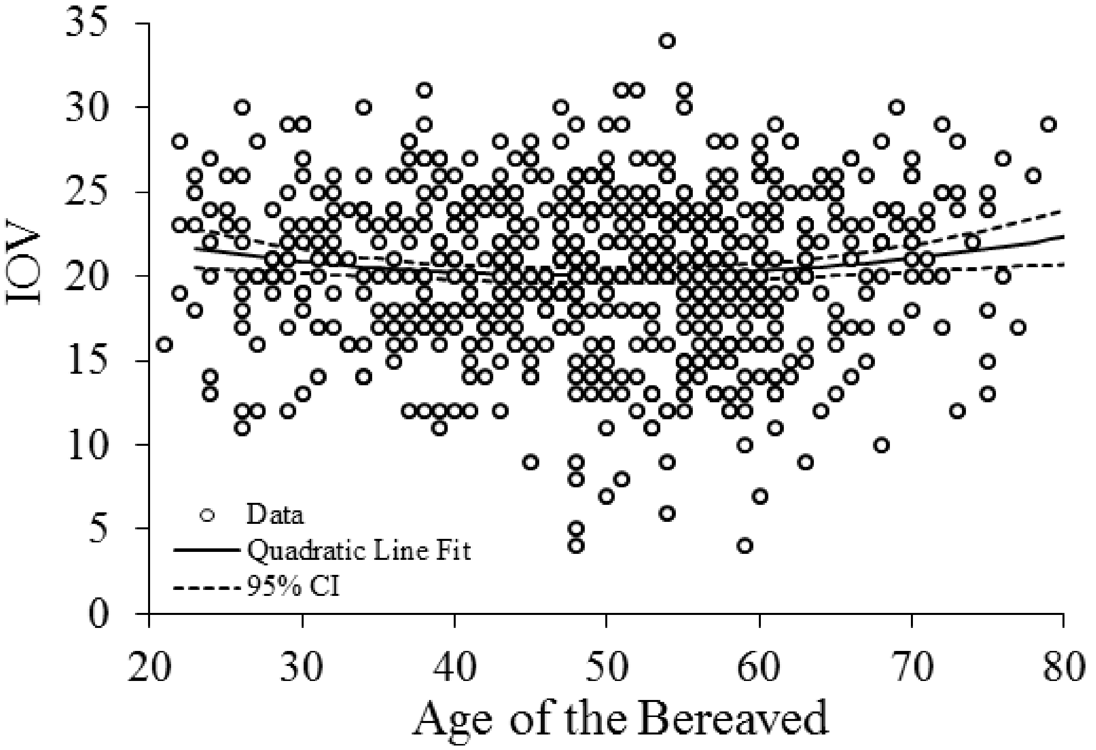

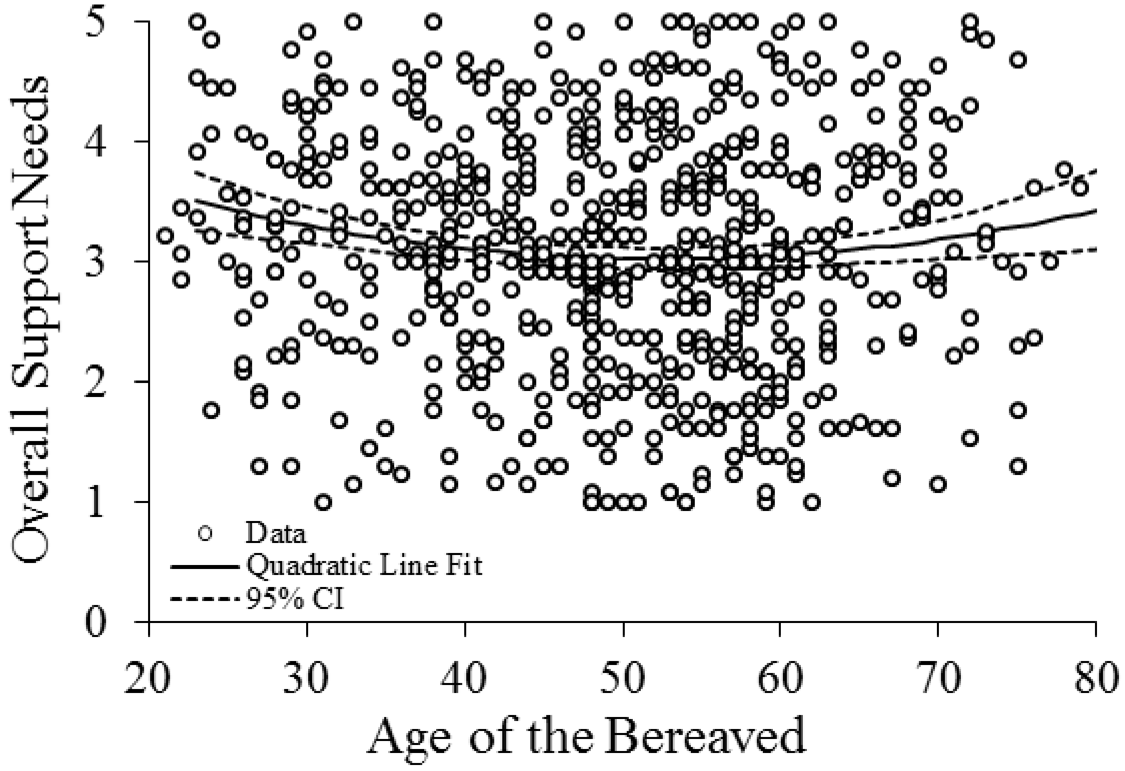

Supplement: Supplemental Material - Factors Associated With Higher Levels of Grief and Support Needs Among People Bereaved During the Pandemic: Results from a National Online Survey [file sj-pdf-1-ome-10.1177_00302228221144925.pdf]
